# Barrelettes without Barrels in the American Water Shrew

**DOI:** 10.1371/journal.pone.0065975

**Published:** 2013-06-03

**Authors:** Kenneth C. Catania, Elizabeth H. Catania, Eva K. Sawyer, Duncan B. Leitch

**Affiliations:** 1 Department of Biological Sciences, Vanderbilt University, Nashville, Tennessee, United States of America; 2 Neuroscience Graduate Program, Vanderbilt University, Nashville, Tennessee, United States of America; University of Lethbridge, Canada

## Abstract

Water shrews (*Sorex palustris*) depend heavily on their elaborate whiskers to navigate their environment and locate prey. They have small eyes and ears with correspondingly small optic and auditory nerves. Previous investigations have shown that water shrew neocortex is dominated by large representations of the whiskers in primary and secondary somatosensory cortex (S1 and S2). Flattened sections of juvenile cortex processed for cytochrome oxidase revealed clear borders of the whisker pad representation in S1, but no cortical barrels. We were therefore surprised to discover prominent barrelettes in brainstem of juvenile water shrews in the present investigation. These distinctive modules were found in the principal trigeminal nucleus (PrV), and in two of the three spinal trigeminal subnuclei (interpolaris – SpVi and caudalis – SpVc). Analysis of the shrew's whisker pad revealed the likely relationship between whiskers and barrelettes. Barrelettes persisted in adult water shrew PrV, but barrels were also absent from adult cortex. Thus in contrast to mice and rats, which have obvious barrels in primary somatosensory cortex and less clear barrelettes in the principal nucleus, water shrews have clear barrelettes in the brainstem and no barrels in the neocortex. These results highlight the diverse ways that similar mechanoreceptors can be represented in the central nervous systems of different species.

## Introduction

The American water shrew (*Sorex palustris*) is a remarkable small mammal that dives into streams and ponds at night as it searches for prey. It is the smallest mammalian diver, typically weighing only 10–12 grams, yet it is an aggressive predator that will feed on virtually any prey item that it can overpower. This includes insect larvae, fish, crayfish, tadpoles, and salamanders. Historically, one of the longstanding mysteries about this species was how it navigates and detects prey, especially given its nocturnal foraging habits [1]. With this question in mind, we previously investigated their behavior, senses, and cortical organization [2]–[5]. We found that water shrews use a combination of olfaction and somatosensation (mediated by whiskers) to detect and pursue prey. Water shrews have one of the shortest reaction times measured for a predatory response, as they will attack a water movement that deflects their whiskers with an onset latency of only 20 milliseconds [3]. In the case of stationary prey, water shrews use their whiskers to rapidly discriminate cast silicone model fish from other shapes [3]. They can also use olfaction underwater by exhaling air onto objects they are exploring and then re-inhaling the same air bubbles [2]. This latter ability allows them to follow a submerged scent trail.

**Figure 1:**
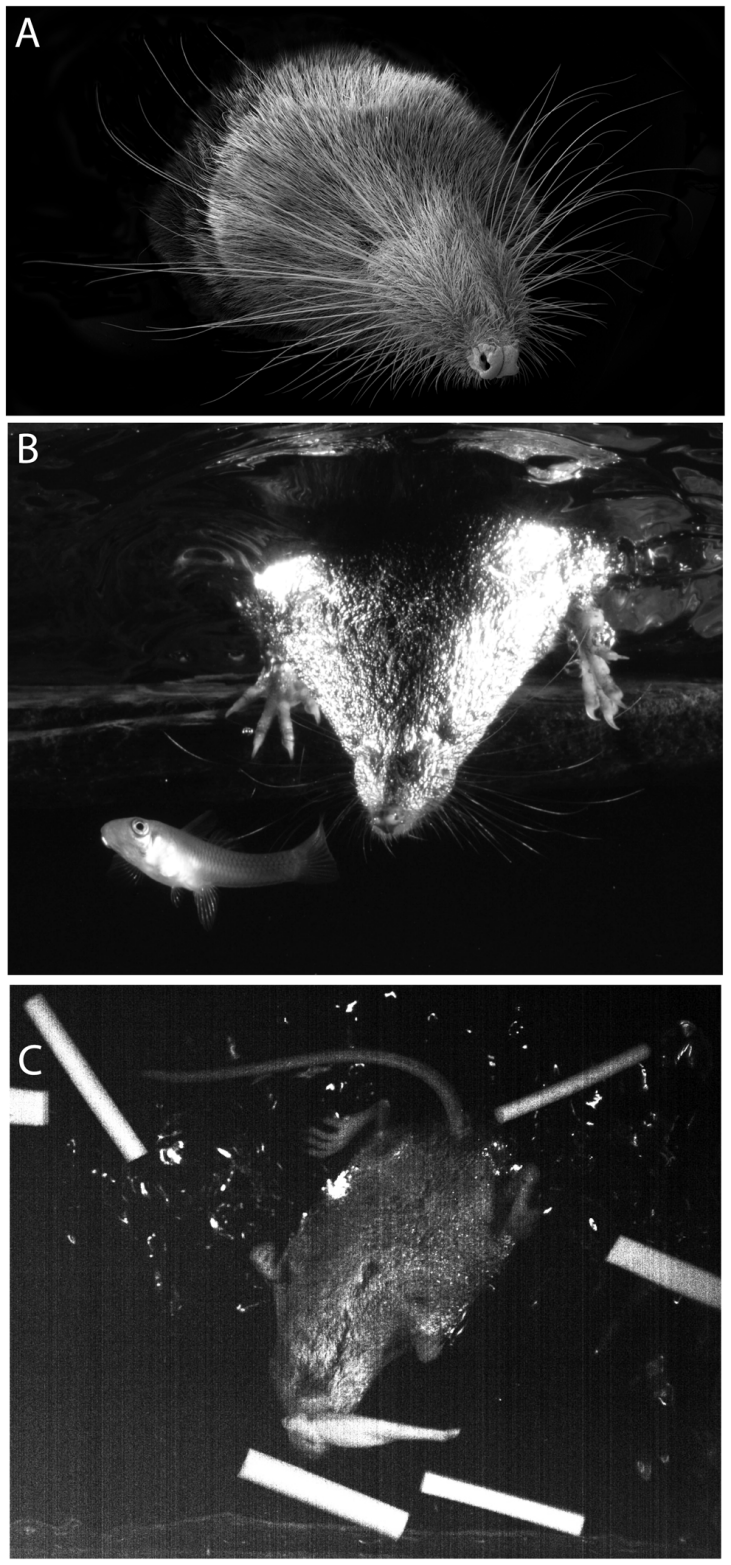
The prominent whiskers of the American water shrew (*Sorex palustris*) guide foraging behavior. A. The water shrew's face under the scanning electron microscope illustrating the prominent whiskers surrounding the nose and mouth. The very small eyes are mostly hidden below the fur. B. A shrew enters the water in pursuit of a quickly moving fish. Water shrews capture moving prey by responding quickly to deflection of the whiskers [3]. Note the large spread of the larger, more caudal whiskers that would likely be deflected as the fish escapes. C. A single frame captured from high-speed video shows a water shrew discriminating a cast model fish from distractors using its whiskers (from [3]). The model was attacked and retrieved. Infrared lighting was used in these trials.

Thus in contrast to the historical view of shrews as being somehow primitive, water shrews rank as one of the more impressive predators in terms of speed and accuracy of sensory discriminations. These abilities depend heavily on the shrew's elaborate whiskers (Figure 1). In contrast, the visual and auditory systems of the water shrew are poorly developed [4]. For example, only 6,300 and 7,000 myelinated fibers supply the shrew's small eyes and ears respectively, whereas the trigeminal nerve contains 27,500 myelinated fibers. These sensory priorities are in turn reflected in the organization of the shrew's neocortex. The primary and secondary somatosensory representations (S1 and S2) are dominated by 2 large representations of the whiskers whereas visual and auditory cortex are both quite small [4].

Despite the behavioral importance of the shrew's elaborate whiskers and the dominance of the whisker representations in the neocortex, we have found no evidence for cortical barrels in water shrews (cortical barrels are the anatomically visible representations of whiskers at the level of the neocortex [6].). The earliest investigations of cortical anatomy were conducted in 5 species of adult shrews, including the water shrew [7]. A later investigation of juvenile water shrew neocortex [4] revealed a distinctive cytochrome oxidase dark wedge of tissue corresponding to the S1 representation of the whiskers, but this region did not contain barrels. This latter result was most conclusive, because juvenile cortex processed for cytochrome oxidase reveals the clearest cortical subdivisions, barrels, and barrelettes whereas in adults such subdivisions may be less obvious [8], [9]. These studies show that shrews have a very different cortical anatomy compared to rodents, raising a number of questions about how whiskers may be differentially represented across species [10].

In the present investigation, we describe a new finding that draws further contrast between rodent and shrew brain organization. The brainstem trigeminal complex of juvenile water shrews contains one of the most impressive sets of barrelettes to be described among mammals (barrelettes are the trigeminal equivalent of barrels – [11], [12]). The shrew's barrelettes were also found in the adult principal trigeminal nucleus (PrV), but in adults even the broad outline of the whisker representation in S1 is attenuated. Thus in contrast to mice and rats, which have obvious barrels in neocortex and less clear barrelettes in the brainstem, water shrews have clear barrelettes in the brainstem but no barrels in the neocortex.

## Materials and Methods

Adult water shrews were collected with Sherman live traps in Northern Pennsylvania under permit COL00087. The animals were housed in Plexiglas cages containing peat moss, sphagnum moss, soil, a water bowl and were fed mealworms, crickets, wax worms, and canned cat food. One female water shrew gave birth to a litter of 5 young. We examined the trigeminal nuclei of 4 juveniles at postnatal days 8, 9, 10 and 12, 3 adult water shrews, and a single C57 mouse (Charles River Laboratories, Wilmington, MA). For histological analysis, each animal was given an overdose of sodium pentobarbital (120 mg/kg) and perfused transcardially with phosphate buffered saline (PBS, pH 7.4) followed by 4% paraformaldehyde in PBS. The brains were removed and post fixed for at least 30 minutes and then cryoprotected in 30% sucrose. One adult brainstem was cut in the horizontal plane (relative to the bottom of the brainstem) and processed as described below. The four juvenile brainstems were cut in the coronal plane. Brainstems were frozen to a sucrose block on the microtome stage, secured with additional 30% sucrose, and sectioned. For two of the four juvenile cases and for the adult cases, the cortical hemispheres were removed from the underlying white matter and subcortical tissue, flattened between glass slides, then cryoprotected in 30% sucrose. The flattened cortex was then frozen, pia side down, to a flat ice block that had been trimmed with a microtome blade to be parallel to the knife edge. Sections of cortex and brainstem were cut at 40 µm, processed for cytochrome oxidase [13], mounted on glass slides, and coverslipped. The whiskerpad of two of the juvenile waters shrews was dissected from the face and flattened between glass slides. It was then dehydrated in a graded ethanol series, transferred to xylene or xylene substitute, and then embedded in paraffin, and sectioned at 10 µm in a direction parallel to the whisker pad surface. Whisker sections were stained for hematoxylin and eosin.

For one adult water shrew, several 2.5 µl injections of (CTB) cholera toxin subunit B (Sigma-Aldrich, St. Louis MO) were made subcutaneously into the middle of the right whisker pad using a 5 µl Hamilton syringe (model 65RN) with a 28 gauge, 1″ needle (model 7803-02) (Hamilton Company, Reno NV) while the animal was briefly anesthetized with isoflurane. Two days later the animal was given a lethal dose of sodium pentobarbital and perfused with 4% PFA in PBS. The brain was removed and the brainstem was separated from the cerebrum. The brainstem was post-fixed in PFA with 4% sucrose for 1 hour and then cryoprotected at 4°C in sequential concentrations of 10%, 20% and 30% sucrose in PBS until the brainstem sunk. The brain stem was then kept in 30% sucrose overnight at 4°C. Coronal sections of 40 µm were cut on a freezing microtome, after which the sections were post-fixed in 4% PFA at 4°C for 4 hours. Free-floating sections were then washed several times in 0.5% PBST (PBS tween), and then washed several times in 2.5% normal rabbit serum (diluted in 0.5% PBST). Sections were incubated in goat anti-CTB (#703, List Biological Laboratories, Campbell, CA) at 1∶4000 in 2.5% normal rabbit serum diluted in 2% PBST for 96 hours at 4°C. After several PBS washes over 30 minutes, sections were incubated in biotin-SP-conjugated rabbit anti-goat IgG (Vector Labs, Burlingame, CA) at 1∶200 dilution in 1% PBST for 2 hours at room temperature. After 25 minutes of washing in PBS, sections were processed using the ABC method (Vector, Burlingame, CA) and rinsed in PBS before visualization by VIP method (Vector, Burlingame, CA). Sections were then washed in dH2O, mounted onto slides, dehydrated overnight and cleared with Citrisolv (Fisher, Pittsburgh, PA) before being coverslipped using Permount (Fisher, Pittsburgh, PA).

Sections were photographed with a Zeiss AxioCam HRc digital camera (Zeiss, Jena, Germany) mounted onto a Zeiss Axioskop microscope using Zeiss Axiovision 4.5 software (Carl Zeiss Microimaging, Thornwood, NY, USA). Sections from SpVi from both sides of one brainstem, and sections from one whisker pad, were imported into *Reconstruct*, version 1.1.0 [14]. Sections were aligned using histological landmarks as corresponding points designated with the stamp tool, and then aligned with the rigid alignment command. Alignment was checked with the blend and flicker commands. The positions of barrelettes and whiskers were then determined and drawn by examining multiple sections. The drawings were exported and placed as jpegs into Adobe Illustrator (Adobe Systems, San Jose, California), and used as a guide to make illustrations shown in the corresponding figures. All procedures conformed to the National Institutes of Health standards concerning the use and welfare of experimental animals and were approved by the Vanderbilt University Animal Care and Use Committee (Animal Welfare Assurance Number A-3227-01).

## Results

### Barrelettes in Water Shrew Trigeminal Complex

**Figure 2:**
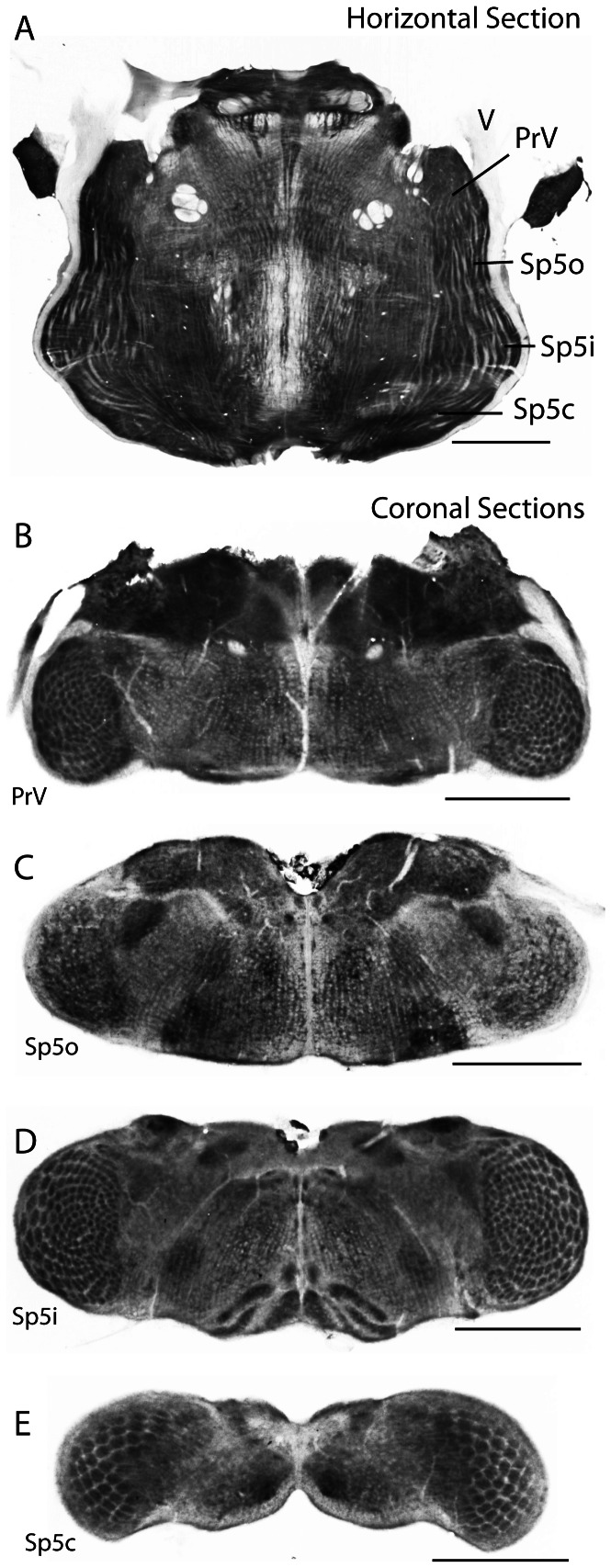
The brainstem trigeminal complex of an adult water shrew. A. A single horizontal section of the brainstem showing the relative size and location of the trigeminal complex. The overall form of the complex is similar to that observed in rodents. The prominent trigeminal nerve (V) enters rostrally (rostral is up in “A”) and the trigeminal tract is evident at the lateral margins of the each complex. PrV, principal trigeminal nucleus. SpVo, spinal trigeminal subnucleus oralis. SpVi, spinal trigeminal subnucleus interpolaris, SpVc, spinal trigeminal subnucleus caudalis. B. A single coronal section from a juvenile (postnatal day 12) water shrew illustrating PrV bilaterally. PrV contained a prominent set of barrelettes. C. A single coronal section from a juvenile (postnatal day 8) water shrew illustrating SpVo bilaterally. No barrelettes are apparent. D. A single coronal section from a juvenile (postnatal day 8) water shrew illustrating SpVi bilaterally. As in rodents, the barrelettes in SpVi were larger and somewhat more prominent compared to those in PrV. E. A single coronal section from a juvenile (postnatal day 8) water shrew illustrating SpVc bilaterally. As in rodents, each barrelette was largest in SpVc. The full pattern of barrelettes was not obvious in single sections through SpVc due to the extreme curvature of SpVc in the coronal plane (see plate A). All sections processed for cytochrome oxidase. Plates C, D, and E are from the same juvenile case, plate B is from a case different from that in C, D, and E. Scales  = 1 mm in all plates. In A, caudal is down, in B–E dorsal is up.

Figure 2A shows a single complete horizontal section through an adult water shrew brainstem, processed for the metabolic enzyme cytochrome oxidase. The brainstem trigeminal complex was visible on both sides just caudal to the entrance of the paired trigeminal nerves (V). As might be expected, the general outline of the shrew's brainstem was similar to that found in rodents [12], [15] with the exception that the shrew's trigeminal complex appeared proportionally shorter in the rostro-caudal direction but wider in the medial-lateral direction. As in rodents, the principal nucleus (PrV) tapered at its rostral extreme and was wider more caudally. As the complex was followed caudally, a slight thinning in the medial-lateral direction marked the oral subnucleus (SpVo) followed by a pronounced widening demarcating the rounded outline of subnucleus interpolaris (SpVi). As in rodents, a light septum visible in horizontal sections marked the border between SpVi and the caudal subnucleus (SpVc).

Coronal sections through four different juvenile brainstems revealed the major finding of this investigation - prominent barrelettes in PrV, SpVi, and SpVc (Figure 2). These had many similarities to their anatomical counterparts in rodents, but also a number of interesting differences. For example, PrV did not have the “peanut” shape that is typical for rodents [12]. Instead it was larger as a proportion of the cross-sectional area of the brainstem and had a hemispherical shape, filled with prominent barrelettes visible in cytochrome oxidase preparations (Figure 2B). In addition, the subdivisions of barrelettes typically seen between the representations of large mystacial whiskers and the microvibrissae [16] of rodents were not obvious in water shrews. Instead, the barrelette pattern was more uniform. Nevertheless, the largest barrelettes (typically corresponding to the largest whiskers in mouse brainstem) were found laterally in water shrews whereas the smaller barrelettes (typically corresponding to the smallest whiskers in mouse brainstem) were found medially in water shrews.

As sections progressed from the more rostral PrV into SpVo, all signs of barrelettes disappeared (Figure 2C). However barrelettes reappeared and were particularly prominent more caudally in SpVi where they were larger than observed for PrV barrelettes (as reported in rodents [12]). As sections continued more caudally, there was overlap of SpVi and SpVc, such that barrelettes from SpVi could be seen ventrally and laterally as columns representing the rostral extreme of SpVc barrelettes appeared dorsally and medially. This progression from SpVi to SpVc was consistent with the orientation of the subdividing septum visible in horizontal sections (Figure 2A), as it indicated that SpVc extends furthest rostrally at its medial extreme.

### Spatial Relationship Between the Whiskerpad and Barrelettes

**Figure 3:**
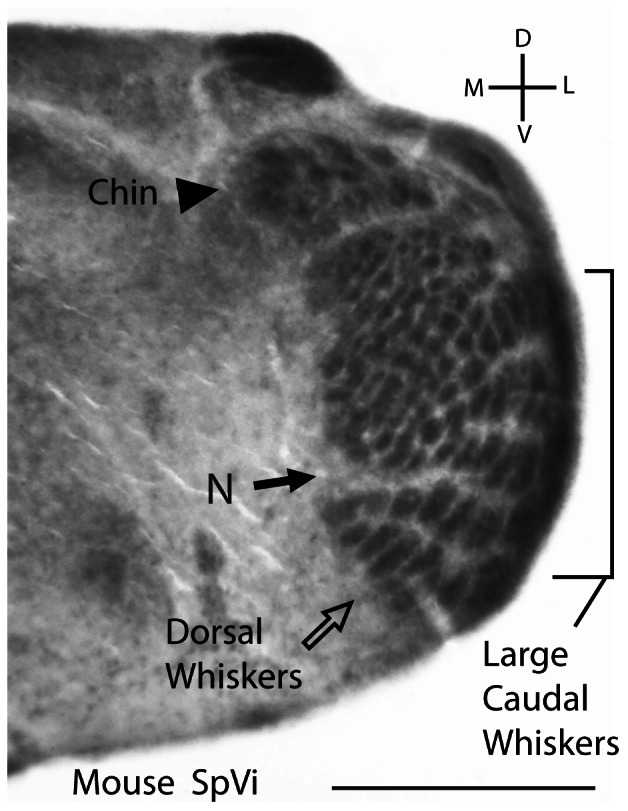
Subnucleus interpolaris from a postnatal day 5 C57 mouse processed for cytochrome oxidase to reveal the barrelettes. The whiskers have an inverted representation with the 5 rows of large whiskers represented ventrally whereas the chin is represented dorsally in a separate module. A small cleft in the barrelette pattern (N) is congruent with the position of the nose on the mouse's face. The designations for chin (filled arrowhead) nose (filled arrow) and dorsal whiskers (open arrow) are used to designate the same parts of the water shrew whisker pad and SpVi barrelettes (Figure 4).

By reconstructing the distribution of whiskers on the sectioned water shrew whisker pad and comparing this distribution to the pattern of brainstem barrelettes, we were able to determine the most likely relationship between the two structures. However, before describing the evidence for this relationship, it is useful to examine the layout of barrelettes in mouse brainstem, as it provided important clues to the condition in water shrews. In both species, the pattern was most pronounced in SpVi, and thus this subnucleus is shown for both species. Figure 3 shows SpVi from a postnatal day 5, C57 mouse processed for cytochrome oxidase to reveal the barrelettes. This well-characterized anatomical representation of the whiskers in mice [11], [12], [17]–[19] illustrates a number of important features of SpVi. For example, the representation of the whiskers is inverted, such that dorsal whiskers on the face are represented most ventrally in the brainstem (open arrow, Figure 3). In mice, there are 5 prominent rows of whiskers and their representation at the level of the primary somatosensory cortex (S1) is often referred to as the “posteromedial barrel subfield”, or PMBSF. These 5 rows are also evident as barrelettes (Figure 3). In mouse SpVi the large caudal whiskers on the face are represented most laterally, whereas the smaller whiskers from the rostral face are represented more medially. Medially and somewhat ventrally in SpVi, a small cleft in the barrelette pattern is congruent with the location of the nose on the rodent's face (filled arrow, Figure 3). More dorsally, the representations of the smaller whiskers of the lower distal face are evident as an array of smaller barrelettes. Finally, at the dorsal extreme of SpVi, there is a slight septum separating the representation of the numerous whiskers on the chin from the remaining barrelettes (arrowhead, Figure 3).

**Figure 4:**
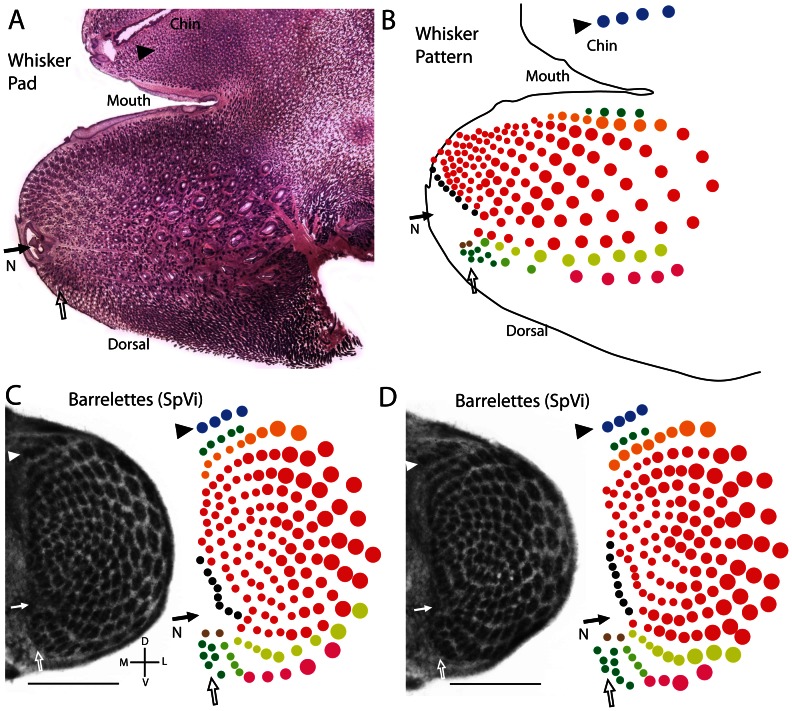
The proposed relationship between whiskers and barrelettes in the water shrew. A. A single section from a juvenile water shrew whisker pad stained for hematoxylin and eosin shows the layout of the whiskers (sinus follicles) relative to mouth, chin, and nose. B. The distribution of whiskers as reconstructed from multiple sections. There were 4 whiskers located on the chin (blue circles). The nose (N), designated with a dark arrow, disrupted the pattern of whiskers providing a landmark reflected in the distribution of barrelettes at the medial extreme of SpVi. A small patch of whiskers was clustered dorsal to the nose (open arrow). C–D. Two examples of the barrelette pattern in SpVi from the left and right side of a single juvenile water shrew. The colored patterns of barrelettes to the right of the tissue sections were produced from examination of multiple sections. The proposed relationships between the landmarks noted on the whisker pad in A and B are indicated in C and D with corresponding colors, arrows, and arrowheads. Note that the whiskerpad illustrated in A and B is from a different case then the histology shown in C and D. Scale bars  = 0.5 mm.

The features outlined above were useful for interpreting the representation of the whisker pad in water shrew SpVi. Figure 4A shows a single (inverted) section of the water shrew whisker pad with important landmarks indicated. Figure 4B shows the full pattern of whiskers relative to the same landmarks. The prominent pattern of barrelettes in shrew SpVi is illustrated for a left and right brainstem in Figure 4 C and D (each from the same animal). As in rodents, the largest barrelettes were located at the lateral extreme of SpVi, whereas smaller barrelettes were located more medially. At the dorsal extreme of SpVi, a short row of 4 barrelettes was evident. This was somewhat different from mice, where a much larger number of barrelettes represent the many small whiskers of the mouse chin. However, it matched the much smaller number of whiskers (four) found on the water shrew's chin (blue circles, Figure 4). Just ventral to this presumptive chin row, there was a short row of small barrelettes restricted to the more medial side of SpVi, adjacent to a more prominent row of larger barrelettes that extended further laterally where the barrelettes became still larger. This also matched the pattern of whiskers on the shrew's face, where ventrally (on the upper lip) a short row of small whiskers lies adjacent to a longer row of more dorsal and larger whiskers (Figure 4B). At the more ventral and medial extreme of the shrew's SpVi, a small cluster of barrelettes was located just ventral to a small cleft in the barrelette pattern. These features also match the pattern seen on the shrew's whisker pad, where a number of small whiskers were located just dorsal to the nose (open arrow, Figure 4 A,B). Thus the nose interrupted the whisker pattern on the shrew's face and this was reflected in the barrelettes (Figure 4, filled arrow), as occurs for mice.

Overall, these features strongly suggest the shrew's whiskerpad maps into SpVi as an inverted representation of the ipsilateral face, as occurs in rodents. The same pattern is evident in compressed form for PrV (Figure 2 and see later figures) suggesting the same pattern holds true for this nucleus as well. However the pattern and distribution of the prominent barrelettes distributed across SpVc is less clear due to the extreme curvature of this nucleus relative to the coronal plane of section (Figure 2A,E).

### Water Shrew Neocortex

**Figure 5:**
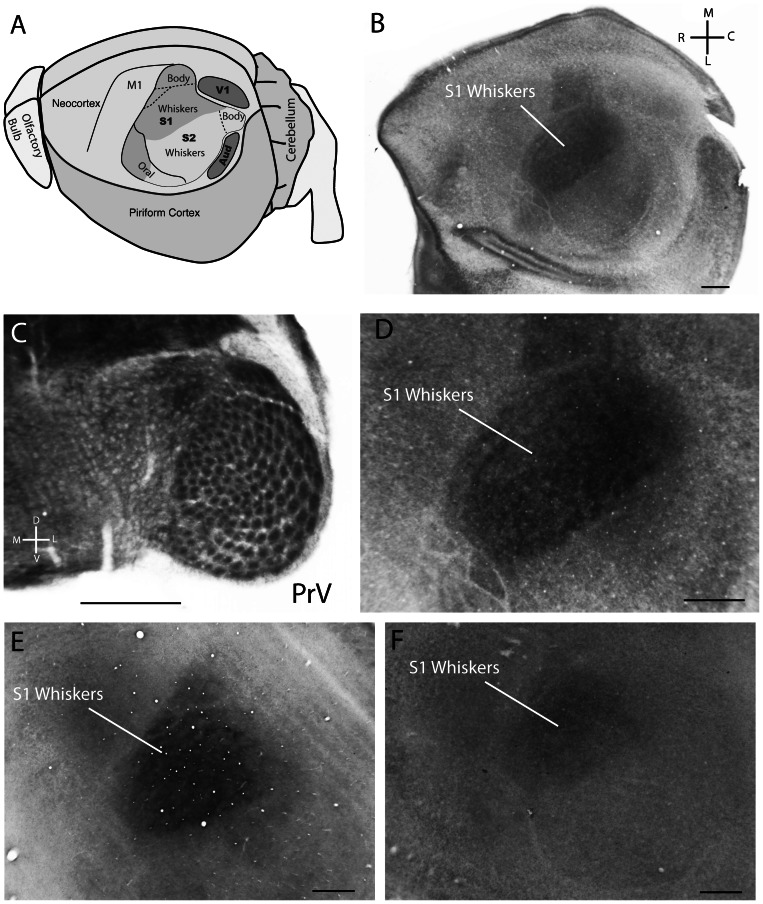
Histology of PrV and cortex from juvenile water shrews. A. The organization of neocortex in the water shrew showing the dominance of the whisker representations in S1 and S2 (after [4]). B. A single flattened section of juvenile shrew neocortex showing the cytochrome oxidase-dense patch of tissue that marks the S1 representation of the whiskers. C. Barrelettes in PrV for the same shrew as illustrated for neocortex in B (section processed for cytochrome oxidase). D. A closer view of the neocortical whisker representation in shown in B. E–F. The flattened neocortex processed for cytochrome oxidase showing the whisker representation, and lack of barrels, for 2 additional juvenile cases. Scale bars  = 0.5 mm.

**Figure 6:**
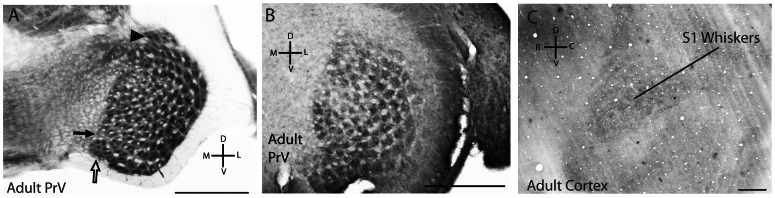
Histology of PrV and cortex from adult water shrews. A. PrV from an adult water shrew (3 months age) illustrating the barrelette pattern. The arrowhead marks the chin barrelettes. Open arrow marks the small patch of barrelettes corresponding to whiskers just dorsal to the nostrils. Filled arrow marks the small cleft consistent with the location of the nose representation (compare to landmarks in figure 4). B. Clusters of afferent terminals labeled by a neuronal tracer (CTB) injected into the whiskerpad of an adult shrew. C. A section of flattened cortex processed for cytochrome oxidase from an adult water shrew (different case from that in “A”). The whisker representation in S1 is visible in the center. Scales  = 0.5 mm.

We have previously investigated both adult and developing water shrew neocortex and reported an absence of barrels [4], [5], [7]. S1 neocortex is illustrated again here for the same case in which prominent barrelettes were evident in PrV (Figure 5). The neocortex of additional juvenile cases is also illustrated. For each juvenile case, the location of the whisker representation [4] was evident as a dark wedge of cytochrome oxidase dense tissue, but no barrels were apparent (Figure 5D–F). In adult water shrews, barrelettes persisted in PrV (Figure 6A) but were less obvious, as occurs for rats and mice. An injection of the neuronal tracer CTB into the whiskerpad of a single adult water shrew revealed a pattern of afferent terminals that mirrored the barrelette pattern in PrV (Figure 6B). This indicated that the cytochrome oxidase dark centers of the barrelettes reflect the predominant location of afferent terminals. Finally, in adult water shrew neocortex, no corresponding barrels were apparent. The broad outline of the S1 whisker representation was still visible, but less pronounced than in juveniles (Figure 6C).

## Discussion

The main result of this investigation was the discovery of prominent, modular representations of water shrew whiskers (barrelettes) in the trigeminal system, despite the absence of corresponding barrels in the neocortex. This result was a surprise, because we had previously investigated water shrew neocortex in adults [7] and also at more favorable juvenile stages for defining cortical subdivisions, and found no cortical barrels [4]. In addition, investigations of the Etruscan shrew cortex found no clear barrel pattern [20].

Patterns of barrels are imparted to the cortex from subcortical levels [21] [21], beginning with the principal nucleus (PrV) [22]. Thus we had assumed that water shrews represent their whiskers differently from rodents throughout the central nervous system, perhaps with less topographic precision than has been widely reported for laboratory rats and mice [6]. Instead, here we find that the water shrew trigeminal system contains some of the most prominent barrelettes reported among mammals. This draws an intriguing contrast between shrews and rodents. Whereas the modular representation of the whiskers is most prominent in the neocortex of rats and mice, it is most prominent in the brainstem of shrews and absent from their neocortex.

It seems important to comment on the developmental stages investigated in the present investigation. We examined water shrew brainstem in the first postnatal week, because this is the most favorable time for visualizing barrelettes in general, and this is also the stage typically examined in laboratory rodents [23]–[26]. In adult mice and rats, barrelettes are less clear (e.g. [12]) and often difficult to identify. Water shrews followed this developmental pattern as well, as the barrelettes were less pronounced in adults. But they were still prominent in adult shrew PrV (Figure 6), the nucleus that provides the main patterning input to the ventral posterior medial nucleus of the thalamus and hence the neocortex [21]. In addition, an injection of the neuronal tracer CTB into the whiskerpad of an adult water shrew revealed clusters of afferent terminals corresponding to the barrelette pattern – further illustrating the similarity between barrelettes in shrews and rodents at the level of the brainstem [12].

Based on the anatomy of shrew cortex compared to cortex in mice and rats, it is possible that shrew thalamocortical fibers (or alternatively trigemino-thalamic fibers) diverge to activate a comparatively large area that encompasses the afferent terminals from adjacent whiskers. Although we have not observed barreloids in the thalamus (barreloids are the thalamic equivalent of barrels) our preliminary investigation of water shrew thalamus is inconclusive because the angle of section to visualize barreloids is not obvious in this species. In any case, wider divergence of projections in the pathways to neocortex could result in a more uniform appearance of the cortical projection zone and perhaps large receptive fields at the level of the cortex. There is some evidence to support this possibility in the form of multi-whisker receptive fields in water shrew S1 [4], [7]. It is also likely that many details of cortical circuitry differ for shrews compared to mice and rats, as the barrels and subdividing septa in the latter receive input from different thalamic nuclei [27] and have different intrinsic and corticocortical connectivity [28].

Given the differences between shrews and rodents, a major question is why shrews have prominent trigeminal subdivisions but lack corresponding modules in the neocortex? There are many possible reasons for the different organizational schemes in different species. Whisking behavior has been previously discussed in the context of cortical barrels that are variably apparent across different rodent species [10]. But results showed that barrels were not clearly correlated with whisking, as demonstrated by barrels in the neocortex of non-whisking guinea pigs [8]. We have not determined whether water shrews whisk. Water shrews are in almost constant motion when awake, and the whiskers exhibit some whisking-like movements when the shrew pauses to explore different objects. But it is not clear whether these are passive movements resulting from abrupt head motions, or active whisking mediated by muscles in the whiskerpad. It will require high-speed videography to investigate this question in more detail.

It could be suggested that whiskers are somehow less important to water shrews and therefore they have a less organized representation in their neocortex compared to mice and rats. But this line of reasoning is not supported by the behavioral data [3]. Water shrews have diverse and impressive predatory behaviors guided by their whiskers. They can track and capture fast moving aquatic prey and can discriminate cast replicas of fish hidden among many distracters using their whiskers. They also have perhaps the fastest documented behavioral response to whisker stimulation [3] as they can direct a bite to the location of a whisker-deflecting stimulus with an onset latency of 20 milliseconds (with only 50 milliseconds for their jaws to reach the location of the stimulus). It is clear from water shrew behavior that the whiskers are important for discriminating prey and guiding rapid and precise head movements while attacking. Thus it is likely water shrews are more dependent on their whiskers than typical for many small mammals.

Perhaps selection for high-speed discriminations and short reaction times has resulted in a greater dependence on the trigeminal system for mediating behaviors compared to the neocortex, which is further removed from the afferent input. This seems compatible with some aspects of shrew biology – for example their rapid muscle contractions and high metabolisms [29]–[31] might require faster neural processing and selection for shorter circuits to mediate behavior. But if this were true, then what is the function of the two large cortical representations of the shrew's whiskers [7]? Another reason to question this interpretation would be the brain and behavior of the star-nosed mole [32]. Star-nosed moles provide an exceptional example of modular neocortex with three different, interconnected cortical maps each containing cytochrome oxidase dark subdivisions representing the different nasal appendages [33]. Despite this complex cortical network of interconnected modules, star-nosed moles are known as the fastest mammalian foragers [32]. It seems likely that fast-acting star-nosed moles depend on their complex somatosensory cortex for sensory processing while foraging, suggesting that water shrews could do the same. However the reaction times measured for water shrews [3] are not directly comparable to the prey handling times measured for star-nosed moles [32] so it remains possible that water shrews have outpaced even star-nosed moles for some aspects of their behavior.

Finally (and not exclusively) it is possible that whiskers can be represented with equal efficiency at the level of the neocortex in very different configurations in divergent species. This possibility is particularly intriguing, given that cortical barrels are often hailed as an anatomical reflection of cortical columns [34]–[36] that have in turn been proposed as a fundamental unit of the mammalian neocortex [37]–[38]. If the occurrence and structure of a fundamental unit of neocortex can vary drastically across species, then we might wonder whether it is truly fundamental [39]–[44].
